# Evidence and argument in policymaking: development of workplace smoking legislation

**DOI:** 10.1186/1471-2458-9-189

**Published:** 2009-06-17

**Authors:** Dorie E Apollonio, Lisa A Bero

**Affiliations:** 1 Department of Clinical Pharmacy, University of California, San Francisco, 3333 California Street, Suite 420, San Francisco, CA 94143-0613, USA; 2 Institute for Health Policy Studies, University of California, San Francisco, 3333 California Street, Suite 420, San Francisco, CA 94143-0613, USA

## Abstract

**Background:**

We sought to identify factors that affect the passage of public health legislation by examining the use of arguments, particularly arguments presenting research evidence, in legislative debates regarding workplace smoking restrictions.

**Methods:**

We conducted a case-study based content analysis of legislative materials used in the development of six state workplace smoking laws, including written and spoken testimony and the text of proposed and passed bills and amendments. We coded testimony given before legislators for arguments used, and identified the institutional affiliations of presenters and their position on the legislation. We compared patterns in the arguments made in testimony to the relative strength of each state's final legislation.

**Results:**

Greater discussion of scientific evidence within testimony given was associated with the passage of workplace smoking legislation that provided greater protection for public health, regardless of whether supporters outnumbered opponents or vice versa.

**Conclusion:**

Our findings suggest that an emphasis on scientific discourse, relative to other arguments made in legislative testimony, might help produce political outcomes that favor public health.

## Background

Workplace smoking restrictions protect workers from the harmful effects of secondhand smoke exposure. [1-3] However, the mechanisms for implementing such restrictions differ internationally and within the United States and may include occupational safety and health regulations,[4] public health department ordinances [5], and legislation instituted at the local, state or federal level.[6,7] These policies can vary in their scope, implementation, and enforcement, and ultimately in their ability to limit exposure to secondhand smoke.[8,9] Moreover, the processes by which different types of restrictions are created vary, suggesting that different factors may influence the development of legislation relative to regulation. For example, legislative decision making can influence regulatory action through actions such as oversight and budgeting, but regulatory agencies do not have similar powers over the legislature.

Past studies of the behavior of regulatory agencies have specifically noted the role of outside testimony in the decision making process, in literature reviewing public commentary provided on two state workplace smoking regulations and the proposed federal Occupational Safety and Health Administration Indoor Air Quality regulation.[4,10-12] In those cases, opposition to proposed regulations came primarily from the tobacco industry, small businesses, and business organizations, and appeared to be coordinated. There was little public health involvement in arguments made for regulation, which is often the case in legislative proceedings as well.[13] Both supporters and opponents made similar arguments, focusing on economic and ideological claims rather than scientific evidence.[4,11,12]

However little empirical research has investigated the ways in which arguments and evidence are employed in the legislative process. Case studies of individual states considering tobacco control legislation showed that scientific debate dominated discussions of proposed legislation in the 1970s and early 1980s. Ideological discourse, particularly arguments about personal freedoms, dominated legislative debates in the mid to late 1980s.[6,14] Later studies showed that tobacco control advocates also began using rhetoric that included ideological arguments about individual rights and innocent victimization, particularly of children.[15,16]

Literature from political science has used qualitative data and case study designs to consider the decision making processes of legislators, particularly at the federal level, both in committee hearings and floor debates. [17-19] Quantitative studies have linked the voting behavior of individual legislators to political ideology, party affiliations, interests of constituents, the views of colleagues, and campaign contributions, among other factors.[14,20-25] However, little is known about the influence of testimony on the policy making process, even though testimony is known to be one of the ways legislators acquire information.[26] Cohen et al (2000)[27] note that in testimony the tobacco industry uses ideological arguments that resonate with "core values" commonly shared by a majority of citizens, and that public health advocates should not ignore these ideological arguments but be prepared to counteract them. Such ideological arguments may be based on general beliefs about the role of government; for example, legislators who oppose tobacco control legislation are typically also opposed to other health promotion policies on the grounds that government should not interfere with individual freedoms.[27] However, among both United States and Canadian legislators, support for tobacco control policies is higher among legislators who recognized or desired more scientific information on the health effects of secondhand smoke.[21,28]

This study examines the relative distribution and type of arguments used in legislative debates at the state level regarding workplace smoking restrictions from the mid-1990s to early 2000s, exploring the nature of legislative debate within and across states using a case study approach that allowed a quantitative analysis of qualitative data. Our findings suggest that greater use of scientific discourse in testimony was associated with stricter workplace smoking restrictions, regardless of whether supporters outnumbered opponents or vice versa.

## Methods

Our study of workplace smoking legislation relied on a case study approach that reviewed the testimony and floor debates in the legislative decision making process regarding workplace smoking restrictions in multiple states, each of which constitutes a case. Our data consist primarily of spoken and written arguments made before and within the legislature and as such are qualitative in nature. However, we were able to identify all of the arguments made in each state included in our study, which allowed us an unusual opportunity to count the types of arguments made within the states we studied. These descriptive statistics do not allow rigorous hypothesis testing, but they do allow a more nuanced comparison of arguments made across states than a more interpretive analysis.

To identify states that proposed clean indoor air laws, we searched the American Lung Association's database "State Legislated Action on Tobacco Issues" and Lexis/Nexis for states that adopted or amended legislation to restrict tobacco use in private workplaces between 1992 and 2002. Our choice of this time period reflects the fact that in 1992, the US Environmental Protection Agency released a report on the effects of secondhand smoke,[29] a triggering event that led several states to consider workplace smoking restrictions in a similar time frame. Using this sampling frame, we identified eleven states that adopted clean indoor air laws and were able to obtain the records of legislative hearings and floor debates for five of these states: Florida, Louisiana, Oregon, South Dakota, and Utah. Although state "sunshine" laws typically require the recording of legislative proceedings, many states unfortunately have not archived recordings for prior sessions; we excluded states that had not archived their records on the grounds that we would be unable to analyze testimony. We identified six states that had proposed but did not pass legislation, but only North Dakota maintained records of its legislative proceedings and, as a result, was the only other case we included.

We collected all available legislative records from the six study sites including audiotapes of committee hearings and floor debates, all versions of each bill introduced, amendments offered during the proceedings, attendance and voting records, any legislative meeting minutes, and public commentary. In addition, in the year prior to the enactment of its first indoor air restrictions, the Utah legislature appointed a taskforce to study the harms of secondhand smoke and to recommend state action. We collected the audiotapes from each taskforce meeting as well as any correspondence submitted. All recordings were transcribed for analysis.

Our analysis proceeded by iteratively coding all oral and written testimony from both legislators and non-legislative participants for each state. If oral and written testimony were submitted by the same participant, the source providing the most extensive arguments was used. Each document was coded for the affiliation of the person submitting it, their position regarding the legislation and regarding tobacco control restrictions generally (e.g., supportive, neutral, or opposed), and the types of arguments made. We developed coding categories for the arguments inductively and based on a coding instrument used in earlier analysis of regulatory proceedings.[4,30] We defined the smallest text unit as a sentence and each text unit could be coded for multiple argument types if multiple categories were applicable. As an example, a statement like "this survey shows that 80% of Oregonians support this legislation" would be coded as both a scientific argument (citing statistics) and an ideological argument (claiming that constituent preferences matter). The broad categories of argument were: (a) science and health effects, (b) economic, (c) ideological, and (d) government/procedural (see Additional file 1 for coding rules and samples of text included under each category). We found that all argument types could be employed in support of or opposition to legislation.

We developed a codebook with decision rules for each category (Additional file 1 provides a definition of each code) to guide the coding process. Three coders, working independently, reviewed all of the documents for each set of argument types. When coding was completed, the full research team reviewed their work as a quality control measure. We used QSR NVivo qualitative computing software for database management and analysis.

To score the strength of state legislation, we applied a modified version of a rating system of state clean indoor air laws developed by the National Cancer Institute  to state legislation included in our study.[1,31] Each proposed law received a summary score based on the extent to which it minimized exposure to secondhand smoke. Two coders, working independently, calculated scores. Any discrepancies were discussed until consensus was reached. The extent of the restrictions was scored in eight workplace categories: government worksites, private worksites, restaurants, bars, retail stores, recreational and cultural facilities, schools, and childcare facilities. We followed the NCI rating system, with each category was scored as smoke-free (4 points); smoking restricted to enclosed, separately ventilated smoking areas (3 points); smoking restricted to areas that are separate or enclosed (2 points); smoking sections or allows exemptions to restrictions (1 point); or no restrictions (0 points). Preemption of stricter local ordinances resulted in a deduction of two points for each affected workplace category with zero as the lowest possible score (our sole modification to the NCI rating system was the application of this preemption deduction only to the relevant workplace category, rather than to the overall score). Points were not deducted from sites already designated as smoke-free. Each piece of legislation was also scored for penalties and enforcement. Points in each of these categories ranged from zero to five points. The total number of possible points for each state law was forty-two. Because the legislation in North Dakota failed, it was assigned a score of zero. Overall, higher scores indicate stronger legislation.

## Results

We begin by summarizing the nature of the cases in our analysis. As shown in Table 1, the six states varied by a range of characteristics (e.g. size of legislature, years in which legislation was considered), as well as the number of outside participants and the strength of their existing or new indoor air legislation. All of the states in our sample considered two to three bills over the course of one or two years with the exception of Florida, which considered six bills over eight years, and as was common during this time period, most states included preemption of stronger local laws in their proposed state laws.

**Table 1:** Study site characteristics (laws passed and in effect)

	Utah	South Dakota	Florida	Oregon	North Dakota	Louisiana
Legislative outcome	passed	passed	passed	passed	failed	passed
Legislative score	33	17	14	12	0(9)*	4
Years deliberated	1993–1994	2001–2002	1985–1992	2001–2002	2001,2003	1992–1993
Percentage of legislators speaking	38%	43%	38%	34%	17%	8%
State legislature size	104	105	160	90	141	144
Non-legislative participants	67	28	42	28	12	3
Preemption	yes	yes	yes	yes	no	yes
Number of coded arguments	753	778	339	560	114	208

*Parenthetical score refers to North Dakota's existing law.

### Utah

In 1993, Utah appointed a task force to study the harms of secondhand smoke. The group was comprised primarily of state legislators, soliciting evidence from many sources, including personal testimony, published studies, and model legislation from other states. The task force recommended that the state strengthen its smoking restrictions, and in its next session the legislature passed a law requiring the separation of smokers and nonsmokers in private workplaces and banning smoking at most other worksites. The majority of the debate centered on the importance of protecting public health and research on secondhand smoke. There was also discussion of the possible economic impact of imposing greater smoking restrictions, and whether Utah would be perceived as excessively moralistic by strengthening its existing law.

### South Dakota

Legislators passed a clean indoor air act in 2002 after debating a similar bill in 2001. The South Dakota law banned smoking in many workplaces and in restaurants that did not serve alcohol. Like all the other states considered in this study, it placed no restrictions on smoking in bars. Debate in South Dakota focused on the health effects of secondhand smoke, protecting public health, and whether passing legislation was necessary to do so. Most of the legislators and many of the outside participants discussed their personal experiences with smoking.

### Florida

Florida passed its first clean indoor air act in 1985. The final bill we considered was adopted in 1992. The 1992 law imposed smoking restrictions by requiring the separation of smokers and nonsmokers in most workplaces and restaurants and continued preempting local governments from adopting policies stronger than the state law. Over the course of eight years, several modifications to the 1985 law were proposed, and most of the debate over these bills referred back to the debate and testimony on the original law. Discussion typically revolved around the importance of protecting public health, with some additional debate on whether businesses could resolve the issue without government intervention, and smoker and nonsmoker rights.

### Oregon

Oregon considered bills in 2001 and 2002 that would restrict smoking in workplaces and restaurants by establishing separate smoking and non-smoking sections. The first bill was signed into law in 2002. It contained a preemption clause that would prevent localities from passing laws stronger than the new state law. The vast majority of the discussion in Oregon revolved around the preemption clause, because many counties at the time were considering legislation stricter than the proposed state restrictions. The importance of protecting the public health was discussed, as were the bills' potential economic impacts.

### North Dakota

In both 2001 and 2003 state legislators in North Dakota proposed strengthening the state's existing clean indoor air act. Although neither bill passed, each would have banned smoking in most workplaces. A large share of the discussion centered on whether or not businesses could meet the demands for non-smoking workplaces without government intervention.

### Louisiana

Louisiana passed bills concerning workplace smoking in both 1992 and 1993. Ultimately, the only smoking restriction implemented was the requirement that businesses post a smoking policy decided upon by their employees. In addition, the laws preempted local governments from establishing policies stronger than state law. The vast majority of the discussion in Louisiana focused on the importance of accommodating smokers with virtually no mention of the adverse health effects of secondhand smoke.

In addition to identifying the numbers of outside (non-legislative) participants in legislative debates, we were able to identify the affiliations of people who testified on proposed legislation and their position on clean indoor air legislation. Unsurprisingly, support for stronger legislation reflected an affiliation with public interests, as shown in Table 2. Tobacco industry representatives uniformly opposed new clean indoor air laws, and 83% of other business representatives, many of which were associated with traditional tobacco industry allies like restaurant and gaming associations,.[5,32] also opposed the proposed laws. In contrast, nearly all government representatives who took a position supported the proposed laws (94%), as did all the representatives appearing on behalf of health interests (such as independent physicians and nurses and the American Medical Association) and non-governmental organizations (like the American Cancer Society). Overall, organizations advocating for collective goals supported clean indoor air laws. However, the distribution of these organizational representatives by state appeared unrelated to the ultimate strength of state laws; North Dakota, which failed to pass a law, had the lowest percentage of participants representing tobacco and business interests (33%) relative to all participants, while South Dakota, which passed one of the strongest laws, had the highest percentage of outside participants representing these interests (43%).

**Table 2:** Support for tobacco control legislation by group affiliation in all states

	support		oppose		no information	total
Tobacco	0%	(0/7)	100%	(7/7)	0/7	7
Business	17%	(10/59)	83%	(49/59)	4/63	63
Other	75%	(15/20)	25%	(5/20)	4/24	24
Government	94%	(17/18)	6%	(1/18)	16/34	34
Health/NGO	100%	(43/43)	0%	(0/43)	1/44	44

Previous research has noted the importance of participation by non-legislators in convincing legislators to enact public health policies.[33] Theoretical research that emphasizes the role of popular participation in decision-making on public goods assumes that increased participation will reflect support for proposed legislation.[34] However, given the relatively low levels of outside participation in debate on clean indoor air laws at the state level, which in the cases we reviewed ranged from 3 to 67 people (including legislative staff members), we considered the possibility that stronger laws might represent disproportionate numbers of supporters appearing before the legislature. Because state legislatures considered a range of laws, some of represented strong clean indoor air laws and some of which did not (Utah, for example, debated passing a law that would have required stricter ventilation standards in lieu of a clean indoor air law), we relied on statements of support or opposition regarding tobacco control legislation in general.

Our results, provided in Table 3 and ordered by the strength of legislation, suggest that the share of supporters or opponents that appeared before the legislature was less relevant than the total numbers of outside participants. Utah, which passed the strongest law and had the greatest number of outside participants, had a smaller share of clean indoor air law supporters (49%) than any state but Louisiana. The number of supporters (20 people) that appeared in Utah, however, exceeded that of every other state but Florida, which debated legislation over a much longer time period. In contrast, advocates for strong clean indoor air legislation in North Dakota outnumbered opponents 2-to-1, but North Dakota, with only twelve non-legislators appearing in total, ultimately failed to pass its proposed legislation.

**Table 3:** Support for tobacco control legislation by outside participants by state

	support		oppose		no information	total
Utah	49%	(20/41)	51%	(21/41)	26/67	67
South Dakota	64%	(18/28)	36%	(10/28)	0/28	28
Florida	59%	(24/41)	41%	(17/41)	1/42	42
Oregon	63%	(15/24)	38%	(9/24)	4/38	28
North Dakota	67%	(8/12)	33%	(4/12)	0/12	12
Louisiana	0%	(0/1)	100%	(1/1)	2/3	3

Note 2: States ordered from strongest to weakest legislation.

Consistent with theoretical expectations about the difficulty of establishing laws that provide collective benefits,[35] amendments to proposed legislation uniformly proposed weakening bills. In many cases, these amendments were offered in response to testimony offered by business and tobacco industry representatives; examples are provided in Table 4. In Louisiana this effect was particularly pronounced; a tobacco industry lobbyist sat in the committee hearings with legislators and rewrote each bill, feeding details of the changes to legislators who officially proposed amendments. The effect was to turn a proposal that would have eliminated workplace smoking into a law that required only the posting of signs regarding the smoking policy determined by individual businesses and that prevented localities from enacting stronger laws. Similarly, in Florida, amendments to bills increased the size of restaurants where smoking restrictions would apply and prevented application of the law to attached bars. However, in states where public health representatives were more visible in hearings (including in Florida in later years), these efforts to weaken proposed legislation in committee hearings were less successful.

**Table 4:** Examples of testimony suggesting amendments to proposed legislation

Florida 1985	SPEAKER: Is it realistic to expect a 50 or a 75 figure to work in the real world?
	MR. JOHNSON [Florida Restaurant Association]: Well, Senators, the logic and the reason that we had asked for the 150 is that that is the dividing line between a restaurant which can get an SRX license to serve cocktails and one which cannot. As such, that is a number target at which architects, restaurateurs, others aim, because they want to get over that 150 threshold. If you look at where the total number of restaurant seats in the State of Florida, which is about 1.8 million is, the majority of those are over that threshold. It's perfectly correct that if you go to a small fast food place like an old style McDonald's or Burger King, though not the new ones, which will tend to be over that threshold too, you know, they will hover somewhere around 50–60 seats. So I guess what I'm saying is to us the logical cut point is 150 because for independent reasons that it is a size limit that is commonly aimed at. If that's not acceptable...
	
	SENATOR VOGT: But that's not before us. We've got a substitute amendment for 75 or an amendment for 100.
	MR. JOHNSON: That's fine.
	SENATOR MALCHON: He said he can live with it.
	MR. JOHNSON: We can live with 75.
	*[elided discussion of amendment phrasing]*
	MR. DICK [Beverage Dealers Association]: I have problems with it, with the 75. I'd like to take cocktail lounges out of it. I don't see any reason to have cocktail lounges in it.
	SENATOR VOGT: That wouldn't bother me either. I assume, you ought to get somebody offer your amendment.
	*[elided discussion of alternate amendment]*
	SENATOR MALCHON: Take the bars out.
	MR. DICK: Thank you, Senator.
	
Louisiana 1992	JO WOOD [Tobacco Institute]: Okay, the next amendment would be on page three, delete after the word, after the word "two"...
	SENATOR LANDRY: What line.
	JO WOOD: Line 2. After the number 2 delete the next sentence, which is line two, three, four and of five, office workplace. And all you're left with then under two is where the employer prohibits smoking in an office workplace, the area in which smoking is prohibited shall be clearly marked with signs. That's all that's going to be left under two.
	SENATOR LANDRY: Okay.
	
Louisiana 1993	SENATOR JOHNSON: So you want smoking to be permitted in those rooms?
	SENATOR LANDRY: Yes.
	JO WOOD: The gaming area.
	SENATOR LANDRY: Just the gaming area.
	JO WOOD: Separate.
	SENATOR LANDRY: The gaming area that's separate.

We considered both the quantity and nature of testimony by specifically coding all arguments made by supporters and opponents. Table 5 provides examples of the kinds of arguments used on different issues; we note that both supporters and opponents used the same classes of argument, and a detailed review of argument types suggested that there were few differences across groups. Outside participants (non-legislators) were more likely than legislators to mention science in their testimony; the percentage of arguments coded as scientific in each state ranged from 1%–35% among legislators, and 0%–62% among non-legislators; non-legislative use of scientific arguments was twice as high as that of legislators in every state but Louisiana and Oregon. These included discussions about research studies, statistics, and the evidence on health effects and exposure to secondhand smoke. The increasing use of evidence appeared to reflect the increasing body of relevant research over time. The content of arguments also differed; supporters of restrictions used arguments about the body of evidence on health effects, secondhand smoke exposure, and protection of worker and public health, while opponents criticized the quality and validity of the evidence. Opponents also referred to studies of the risks of secondhand smoke as being "fundamentally flawed," "scientifically unsound," and "invalid." Regardless of their affiliation, opponents discussed the loss of business and the cost of compliance whereas supporters emphasized savings in health care costs and worker productivity. In later years, supporters also cited peer reviewed studies that refuted opponents' claims that there would be negative economic impacts on businesses became smoke-free.

**Table 5:** Examples of arguments used in legislative debate

Supporters	Opponents
*Ideological (rights and freedoms)*	
"We don't question that people have the right to smoke. The key issue is whether the smoker has the right to deny to the non-smoker access to a healthful, pleasant, safe environment."	"The fact remains that most Americans, smokers and non-smokers alike are weary of tighter government regulation on any issue."
"And, it is not so much a right of a person to smoke, as a right of the public to be protected from that smoke."	"Colleagues, this is not a bill about health. This is a bill about control.... This is another example of this body, the big brothers and the big sisters in [the state capital], telling those little towns and those little counties what it is we are going to let them do or not let them do."
*Scientific evidence*	
"There are studies well documented, and documented in some of the most prestigious medical journals we have, indicating that lung cancer is definitely increased in the spouses of smokers... Studies in children over and over have documented chronic lung disease... Pediatric literature is filled with report after report in regard to documentation in regard to this."	"But I don't believe these statistics. I mean I don't think they are statistics. I think they're just numbers. I think statistics are proven with scientific evidence that passes a variety of tests... But I think for some people to say 3,000,000 of this or 100,000 of that and I think they believe it, but I don't think it's scientific."
"This is called Up-to-Date. This is a computer program that I ran last night on medical research. It's got articles from 270 English speaking journals around the world for the last 12 years. This represents a synopsis of all the data that we know about secondhand smoke and it's compiled and it's up to date as of yesterday... There's [sic] nearly 100 references. Over and over and over again it talks about areas where we have secondhand smoke as a problem."	"The agency document is long, imposing and carries the weight of the federal government. However, it is a study with distorted guidelines, lowered statistical standards, unjustified claims of certainty and incomplete and biased selection of data from the literature. The document is not good science."
*Economics*	
"A smoking employee costs the employer at least 1,000 dollars per year in total excess direct and indirect health care costs, compared with a similar nonsmoking employee."	"The legislation will be difficult to enforce and will place an unfair burden on employers, proprietors, etc., required to enforce it. The law may lead to confrontations with employees, patrons, and others which will be disruptive and perhaps costly."
"Independent, objective, and peer-reviewed studies from across the country have demonstrated that there is NO negative impact on restaurant sales or employment from smoke-free restaurant laws. Studies indicate that the impact of smoke-free laws and ordinances do not adversely affect, and may increase business."	

**Figure 1:**
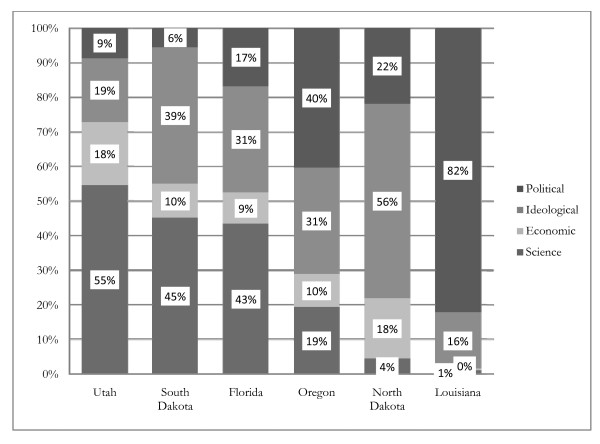
**Distribution of argument types and strength of legislation across states**. Note: States ordered from strongest to weakest legislation.

These results suggest the importance of both participation in the process and a focus on relevant public health evidence. Our coding of the arguments presented in both committee hearings and floor debates allowed us to match the types of arguments made (where each mention of a particular issue counts as an instance of that argument) with the states and the strength of legislation. Our findings, which are presented in Figure 1, show the proportions of arguments in legislative debates in each state relative to the strength of the law ultimately passed (or retained, in the case of North Dakota). We note that the states where arguments primarily related to scientific evidence about the risks of secondhand smoke exposure passed stronger legislation. The differences in this distribution of argument types, specifically the more extensive use of scientific arguments in states with stronger legislation, were statistically significant (Pearson's correlation coefficient = 0.921; two tailed p-value < 0.01). Although it is difficult to disentangle the role of participation in the legislative process from the arguments made given the nature of our research design, our findings suggest that an emphasis on scientific research in the policymaking process, like increased participation, is associated with stronger public health legislation.

## Discussion and conclusion

Our analysis of the participation and arguments employed in legislative debate on workplace smoking legislation suggests that the greater the proportion of arguments focused on science and health effects, the more common it was to see favorable public health outcomes, independent of the affiliations of outside participants who testified on proposed legislation. A key factor in passing public health legislation may be the tacit agreement among participants in the policy making process that a particular policy question is one that should be informed by scientific evidence rather than based exclusively on political, economic, or ideological concerns. Although elected officials are responsible for representing constituent preferences regarding these and other factors, these may not be definitive in all cases, and the pursuit of effective policy may require reliance on scientific evidence, including research on effectiveness that may indicate that one policy is a better means of reaching a particular outcome than another.

Consistent with earlier studies of regulation and legislation development, the main opponents to public health legislation were business interests, while health and government representatives made up most of the supporters.[4,13,36,37] However, the distribution of these interests does not appear to have been relevant. Our findings suggest that legislators may view testimony in committee hearings as a simplified sampling mechanism for popular opinion, and that achieving a critical mass of supporters at legislative deliberations may be more important than whether these supporters are outnumbered by opponents. This finding is consistent with expectations that legislators discount self-interested testimony, [38-43] in this case, protestations by the tobacco industry and other business representatives that the laws would impose economic costs exceeding the public health benefit. One proposed strategy for increasing the success of public health legislation is to encourage and facilitate public health participation in the legislative process.[33] As Cohen and Jacobson have suggested, this could be accomplished by convening taskforces of scientific experts, as was done in Utah, or by obtaining financial support that would allow public health advocates to testify.[13,27] State-university partnerships, including one pioneered with the California Health Benefits Review Program, which provides independent analysis of the medical, financial, and public health impacts of proposed state health insurance mandates, could also facilitate the use of scientific arguments in legislative debate.[44]

Although outside participants in the legislative process used economic, ideological, and political arguments in almost all states, such arguments made up a smaller share of the debate in states that passed stronger legislation. One strategy proposed to combat the ideological arguments made by those opposed to public health legislation has been the promotion of ideological counter-arguments.[27,45] This strategy contrasts with the alternative approach of emphasizing scientific discourse in legislative debates, a tactic commonly used in efforts to pass public health legislation from the 1970s to the early 1980s.[6,14] Our findings, although based on an assessment of the nature of arguments presented in testimony across a limited number of cases, suggest that states where legislative discussion focused primarily on scientific evidence passed stronger public health legislation. Although our research design does not allow us to control directly for factors other than the testimony such as state political culture, the widely varying use of such scientific argumentation across states–particularly states such as North and South Dakota, which have similar political cultures but where legislative debates varied enormously in the share of arguments devoted to scientific evidence and resulted in very different outcomes–suggest that advocacy strategies emphasizing scientific discourse deserve renewed consideration. That said, a useful expansion of this research would investigate the role of other factors that vary across states and appear to influence legislative decision making, including the level of lobbying, extent of campaign contributions made by industry, and the role of affected constituencies within the state (e.g. tobacco growers, cigarette manufacturers). Nonetheless, in the states that passed stronger legislation in our study, more discussion of science appeared to be associated with laws that provided greater protection for public health regardless of whether the discussion was critical or favorable. Moreover, the discussion of science, as coded in this study, was rarely sophisticated; many participants simply introduced the idea that there were studies to refer to on the topic. Further study of testimony could help public health advocates identify the extent to which they should concentrate on different types of arguments.

Analysis of the arguments used in legislative debates helps elucidate the beliefs of supporters and opponents of public health legislation. As predicted by Cohen, both use arguments that appeal to core values about rights, freedom and personal liberty.[27] However, adherence to these core values appeared unrelated to legislative outcomes. When discussion focused more on scientific evidence than other types of argumentation, public health outcomes prevailed. Our findings suggest that more scientific discourse may help generate policy outcomes that are aligned with the strong evidence that secondhand smoke is harmful; further study might consider whether this finding could be relevant to other public health issues.

The study has several limitations. First, we cannot establish a cause and effect relationship between the frequency and content of arguments in legislative debate and the policy outcome. Furthermore, the underlying social norms of states can influence policy; American states have different political cultures, existing restrictions, legal constraints on legislators (such as term limits), staffing levels, and may have part-time or full-time legislatures. States with a long tradition of supporting the interests of business and with weak campaign finance regulation, such as Louisiana, may be resistant to passing clean indoor air legislation regardless of the extent of scientific discussion. Second, our comprehensive analysis of the public record did not allow us to evaluate the potential impact of ex parte or non-official communications among participants in the legislative process. However, the analysis of public hearings and commentary is an important vehicle for describing and understanding the framing of policy issues and the types of evidence and argument that are considered. Finally, states with part-time legislatures that meet irregularly may not have the time to engage in much substantive policymaking. However, certain consistent themes related to the type of argument emerged when we conducted detailed examination of individual state cases where both context and outcomes differed dramatically.

Our findings suggest strategies for supporting future public health legislation. First, public health advocates and their legislative allies should try to frame the policy debate by making reference to research that supports public health goals. Second, public health advocates may wish to experiment with the mix of ideological counterarguments and scientific evidence they present in the limited time available for public testimony to determine whether emphasizing different types of arguments affects their perceived credibility and effectiveness. Overall, our findings suggest that a renewed emphasis on scientific discourse in legislative testimony may help produce political outcomes that favor public health.

## Competing interests

The authors declare that they have no competing interests.

## Authors' contributions

DA collaborated in the study conception and coding, completed the analysis, and drafted portions of the manuscript; LB collaborated in the study conception, drafted and reviewed portions of the manuscript, and supervised the research project. Both authors read and approved the final manuscript.

## Pre-publication history

The pre-publication history for this paper can be accessed here:



## Supplementary Material

Additional file 1 **Appendix: Excerpts from the codebook**. Excerpts from the codebook Click here for file
