# Supplementary material for: Evidence and argument in policymaking: development of workplace smoking legislation
Source: BMC Public Health. 2009 Jun 17;9:189. doi: 10.1186/1471-2458-9-189 (PMC2706247; doi:10.1186/1471-2458-9-189)
Supplement: Additional file 1 — Appendix: Excerpts from the codebook. Excerpts from the codebook [file 1471-2458-9-189-S1.docx]

# Appendix: Excerpts from the codebook

## 1. Scientific arguments

### 1.a. Statistics

Code the presentation or description of statistical information, including any mention of numerical data. Statistical evidence may include terms related to statistical analyses such as: t-test, p-values, or confidence intervals. Also code argumentation such as: “statistics show” or “statistics prove.”

Examples:

- “Eighty-five percent of responding firms have smoking policies designed to address employee health and comfort, up from 54 percent in 1987 and 36 percent in 1986.”
- “The US EPA document… used… questionable statistical manipulations…”

### 1.b. Citations to studies

Code statements that refer to specific sources or studies and that are cited as evidence in an argument. Typically used to code an individual study, the statement might make reference to an author, a journal, or a common name of a study. Include all citations, even if they refer to questionable or unscientific sources such as newspapers, editorials, and industry reports. Also code statements referring to peer review.

Examples:

- “EPA ETS risk assessment shows…”
- “Dr. Hales said the NIOSH study shows…”

### 1.c. Air Quality-Ventilation

Code statements that refer to research on air quality or ventilation. Include arguments that a secondhand smoke problem can be solved or not solved by ventilation and comparisons to other compounds. Arguments may include terminology such as “toxic fumes,” “particulate matter,” “air quality standards,” “ASHRAE,” “air exchange rates.”

Examples:

- “…presented an overview on ventilation and procedures to obtain acceptable air quality from ETS [environmental tobacco smoke]…”
- “The ASHRAE 62-1989, Ventilation for Acceptable Indoor Air Quality Standard, does not and cannot ensure the avoidance of all adverse health effects, but it reflects recognized consensus criteria and guidance.”

### 1.d. Exposure

Code statements referring to actual or hypothetical exposures (e.g., children may be exposed in bowling alleys) or claims that a level of exposure to secondhand smoke is sufficient/insufficient to result in health effects. In general, code statements referring to a dose or exposure. Do NOT code when used only as a qualifier of toxicity (e.g., exposure to secondhand smoke causes cancer). Consider whether the meaning is changed at all by the elimination of the word exposure (e.g. secondhand smoke causes cancer).

Examples:

- “A recent publication estimated the typical SHS [secondhand smoke] exposure of the U.S. population to be around the equivalent of a single cigarette per year.”
- “Actual exposures to SHS in the workplace are low and therefore not likely to produce any health effects.”

### 1.e. Adverse Health Effects-Medical Conditions

Code statements referring to health effects or medical conditions attributed to smoking, exposure to secondhand smoke, or to comparison cases.

Examples:

- “SHS [secondhand smoke] exposure has been shown to be associated with a multitude of health effects including lung cancer, respiratory and heart disease.”
- “We are one of the last states in this nation that has dealt with the problem of smoking. And we have more lung cancer, and more people dying from cancer in this state, than just about any other state in the nation.”

### 1.f. Quantity-Critical Mass of Scientific Evidence

Code mentions of the fact that there is/is not a critical mass of scientific evidence to support a given position, including arguments of unsupported conclusions or the call for more evidence.

Examples:

- “There is/is not a sufficient amount of evidence to support this regulation/conclusion”
- “The weight of evidence is not sufficient”
- “Evidence of causality is sufficient.”

### 1.g. Consistency

Code mentions of the fact that the body of evidence presented to support a given position repeatedly came to the same conclusion, or failed to reach a consistent conclusion.

Examples:

- “Even studies using different methodologies to address the same question came to the same conclusion.”
- “Although not always statistically significant, the results of most epidemiological studies of SHS [secondhand smoke] exposure and lung cancer consistently demonstrate an elevated risk of lung cancer”

### 1.h. Quality-Scientific Merit

Code mentions of judging the body of evidence based upon an evaluation of its merit, especially publications in peer-reviewed journals, and assessments of analyses done by scientific advisory boards. Include methodological critiques and arguments of outdated or irrelevant material and flawed studies.

Examples:

- “This investigation, funded by the National Cancer Institute, was the largest study ever conducted and incorporated most of the state-of-the-art controls to address confounding variables.”
- “EPA’s analysis suggests that the authors of the risk assessment were well aware of the weaknesses of the SHS [secondhand smoke] epidemiology and in fact were able to support their conclusions only by relaxing the statistical standard used to evaluate the data.”

### 1.i. Validity

Evaluations of scientific evidence in terms of the degree to which the measurements truly reflect the concepts or phenomena they were intended to measure. Discussions of “biomarkers” and appropriate and inappropriate measurements would be coded here. Include arguments of whether scientific evidence can be generalized.

Examples:

- “I’m not sure one can accurately measure cotinine levels using saliva.”
- “The ETS risk assessment is not a workplace study, but is based instead solely on studies of nonsmokers assumed to be exposed in the home. Accordingly, claims that the report supports smoking bans in public places are totally without scientific foundation.”

## 2. Economic arguments

### 2.a. Cost to Individual Businesses-Employees

Arguing for or against the proposed legislation on the basis that businesses (or employees) will make more (or less) money as a result of individuals increasing (or decreasing) their patronage of particular establishments. Include contentions that if a smoking ban is in place, individuals, industries, businesses, or conventions would relocate to areas that do not have smoking bans. Also, without consistent regulation, patrons will travel to areas that do not have smoking bans.

Examples:

- “We need a level playing field.”
- “If you ban smoking in restaurants the majority of diners, who are nonsmokers, will stay longer, eat more, and leave better tips.”
- “If you ban smoking in casinos, smokers will simply stay home.”

### 2.b. Cost to Government

Arguing for, or against, the regulation on the basis that it would cost the government (local, state, federal) more or less, to have or not have, the smoking ban.

Examples:

- “The smoking ban will cause the city to lose hospitality tax revenues.”
- “The smoking ban will save Medicare and Medicaid money.”

### 2.c. Cost-Benefit to Employer-Employee

The proposed regulation/legislation will affect employer/employee relations in the workplace. The smoking ban will help or hinder the employer, such as increase or decrease productivity, lose or gain profit, help or harm insurance premiums, etc.

Example:

- “The regulation will help stem the tide of the workmen’s comp crisis.”

### 2.d. Cost of Compliance

Code any statements regarding the cost (or the lack of cost) of complying with smoking restrictions.

## 3. Ideological arguments

### 3.a. Protect the Health of Workers

Workplace smoking bans protect workers’ health as well as making it possible for those with smoke-aggravated illnesses to work.

### 3.b. Protect the Health of the Public

Workplace smoking bans protect customer’s health. This claim may be made in the Americans with Disabilities Act (ADA) sense: smoking bans in public places are a reasonable accommodation for individuals whose health is compromised by smoke.

Examples:

- “We need to protect children and adolescents who are unable to make informed, conscious, adult decisions.”
- “Parents can freely choose whether or not they want to expose their children to smoke in a restaurant. Children’s health is a parent’s responsibility.”

### 3.c. Rights-Freedom

Asserting that smokers have the right to smoke, or nonsmokers have the right not to inhale secondhand smoke. This category encompasses discussions of fairness and equality. Smokers or nonsmokers should not be distinguished as different (i.e., second class citizens) and oppressed for smoking or not smoking.

### 3.d. Privacy-Governmental Intrusion-Free Enterprise

Government should not intrude into private enterprise.

Examples:

- “We need less regulation—there’s too much red tape already!”
- “Regulatory overkill”
- “If a problem exists market forces will correct it!”

### 3.e. Constituent Preferences

Arguments about the proposed ban that state the direction of public opinion.

Examples:

- “The majority of the citizens of Oregon support this bill.”
- “Our constituents support this law.”

### 3.f. Prohibition

Legally forbidding smoking is an extreme action, destined to fail as did the 18th amendment. This code also includes claims of extremism or a “slippery slope”.

Example:

- “A workplace smoking ban will open the door for a no-drinking policy, and then a no-eating-red-meat policy.”

### 3.g. Courtesy-Mutual Respect-Accommodation

Nonsmokers should be respectful of smokers. Smokers should be respectful and considerate of nonsmokers by not smoking around them. Setting up ventilated smoking areas is/is not a reasonable accommodation for smokers.

## 4. Government-Procedural arguments

### 4.a. Citizen Confusion

The claim that in the absence of a uniform standard, individuals who travel between cities or counties within the state will be unable to keep track of the smoking laws as they vary from city to city or county to county and thus may inadvertently break the law. Or conversely, citizens have no trouble discerning laws in different locations when they are clearly posted.

Commonly used terms:

- “uniform standards”
- “patchwork”

### 4.b. Jurisdictional issues

The argument that city or county officials are closer to the people and are more informed about what they want. Alternatively, making the claim that cities or counties have a right to police themselves. Also claims that preemption would wipe out existing city/county ordinances or prevent cities/counties from passing future ordinances. Conversely, cities/counties can't be trusted to be reasonable. In addition, arguments regarding whether or not one entity (e.g., the state board of health) has the legal ability to regulate in this area coded as a “vertical” jurisdictional claim (city versus state).

### 4.c. Existence-Absence of Worker Protection Law

A statewide standard is needed to protect thousands of workers currently unprotected. Or conversely, workers are already protected under other state law(s).

### 4.d. Enforcement-Implementation

Claims that attempt to predict the ease or difficulty of implementation or the effectiveness of enforcement mechanisms, especially with respect to employer compliance. Also, discussions of whether or not the designated enforcement agency is a good choice – at either the state or local level (but not between levels or branches). Also includes arguments on the adequacy or inadequacy of existing enforcement; concerns over who should be designated responsible for building, business, or office compliance; and any discussion of management, authority, designation at the level of the site of employment.

Examples:

- “This regulation is going to be an enforcement nightmare—we are turning employers into police.”
- “Enacting this regulation will take the onus off of employers.”

### 4.e. Procedure-Process

Discussions regarding the process of developing the legislation i.e., how did the committee determine what evidence should be considered, who determined who should testify, claims that there is not an equal balance of “pro” and “anti” tobacco people testifying, claims that the committee chair is not following proper procedure or guidelines.

Example:

- “What is this bill doing in this committee? It should have been assigned to the Health and Welfare committee.”

### 4.f. State Power to Regulate

Arguments claiming that the state does or does not have the authority to regulate issues of public health.
